# Prevalence of Biomarkers and Associated Factors for Chronic Kidney Disease in Adult Diabetic Out-patients in a Tertiary Hospital in Eastern Uganda - a Cross-sectional Study

**DOI:** 10.21203/rs.3.rs-3992049/v1

**Published:** 2024-03-05

**Authors:** Moses Kirya, Denis Bwayo, Michael E. Otim, Paul Bukhota Mutoo, J.Peter M. Masaba, Ambrose Okibure, Richard Katuramu

**Affiliations:** Busitema University; Busitema University; Dubai Medical College; Busitema University; Busitema University; Makerere University; Busitema University

## Abstract

**Background:**

Chronic kidney disease (CKD) is one of the most common complications of Diabetes Mellitus (DM). DM contributes to about 66% of CKD cases globally. CKiiiD results in increased morbidity and mortality and advanced stages often require renal replacement therapy that is unaffordable for the majority of the patients. Developing countries have scanty data regarding CKD burden in diabetic patients.

**OBJECTIVES:**

This study aimed at determining the prevalence of biomarkers for CKD and associated factors among diabetic patients attending the adult diabetic clinic of Mbale Regional Referral Hospital (MRRH).

**Methods:**

A cross-sectional study was conducted at the adult diabetic clinic of Mbale Regional Referral Hospital in Eastern Uganda. 374 adult diabetic patients who consented, were recruited and interviewed. A urine sample for Urine Albumin Creatinine Ratio (UACR) determination and a venous blood sample for measurement of serum creatinine were obtained from each participant. The estimated glomerular filtration rate (eGFR) was determined using the CKD-EPI equation and CKD was staged according to the Kidney Disease Improving Global Outcomes (KDIGO) systems.

**Results:**

A total of 318 (85%) participants had an eGFR of ≤ 60mls/min/1.72m^2^, significant proteinuria, or both. 6.1% were aware. Age, Duration of DM, Hypertension, and Dyslipidemia were associated with CKD biomarkers.

**Conclusion:**

There is a high prevalence of biomarkers for CKD among DM patients, the majority of them being undiagnosed. Over half of the DM patients had an eGFR consistent with advanced CKD. Strengthening routine screening for CKD biomarkers and enhancing the DM clinics with more diagnostic resources is recommended.

## INTRODUCTION

Chronic kidney disease (CKD) is currently a major public health challenge worldwide, with an estimated global prevalence of 9.1% (700 million cases) according to the World Health Organization (WHO) Report of 2023. The global burden of CKD has increased by about 29.3% since 1990. According to the Global Burden of Disease (GBD) study 2017, CKD was the 12th leading cause of death globally - an increase from the 17th in 1990. The most common cause of CKD is Diabetes Mellitus (DM), which has also been on the increase [2]. The International Diabetes Federation (IDF) estimated that the number of adults with diabetes will expand by 55% - an increase from 381.8 million (in 2013) to 591.9 million in 2035. The largest increase is estimated to occur in Sub-Saharan Africa (SSA) [3]. About 30–50% of patients with DM develop manifestations of diabetic nephropathy in their lifetime [3]. In SSA, the prevalence of DM nephropathy is estimated to be 11–83.7% [3,4,5].

The risk of developing CKD among diabetic patients is influenced by the duration of diabetes, hypertension, advancing age, obesity, and poor glycemic control [8,9,10,11]. It is also more common in least-developed countries compared to developed countries and this is mainly attributed to; delayed diagnosis, limited screening and diagnostic resources, and poor glycemic, among other risk factors [3]. This late detection in turn leads to inadequate treatment of CKD at an early stage in such populations and a higher burden of advanced disease with poorer outcomes. Early detection of CKD in diabetic patients is associated with improved care to the patient and delay of progression of kidney disease. This relies largely on the detection of certain biomarkers that are associated with kidney disease. The American Diabetes Association, Kidney Disease Improving Global Outcomes (KDIGO), and the National Kidney Foundation (NKF)’s Kidney Disease Outcomes Quality Initiative (KDOQI) recommend the use of the estimated Glomerular Filtration Rate (eGFR) and the Urine Albumin Creatinine Ratio (UACR) as the tests for early detection of CKD especially among the major risk groups which include diabetic and hypertensive patients [1]. The combination of these two, eGFR and UACR guides therapeutic interventions as well as helping to predict the risk for CKD progression plus cardiovascular events, and mortality.

The evidence to guide screening programs and thereby address the burden of kidney disease among diabetic patients in Africa remains scanty [3]. Studies carried out in Uganda about CKD show that the prevalence is about 15.2% [14]. However, this was carried out in the general population in an urban setting not in a DM population in particular. The paucity of local evidence regarding the prevalence of CKD among diabetic patients in developing countries where Uganda is inclusive creates challenges in designing locally tailored prevention and management programs. This knowledge gap also contributes to the reluctance to incorporate screening for CKD biomarkers in the routine care of diabetic patients in our setting which partly contributes to the delayed detection.

Therefore, our study aimed to determine the prevalence of biomarkers for CKD and associated factors among the adult diabetic patients seeking care in the adult diabetic clinic of Mbale Regional Referral Hospital in Eastern Uganda.

## METHODS

A descriptive cross-sectional study was conducted between December 2022 to May 2023.

### Study site

The study was conducted in the Mbale Regional Referral Hospital (MRRH) adult diabetic outpatient clinic. It is located in the Eastern part of Uganda, within Mbale City, which is 222 kilometers from the Capital City - Kampala. The diabetic clinic runs within the MRRH outpatient clinic and is one of the 16 Regional Referral public health facility treatment centers across the country. The MRRH is the main referral hospital in the Eastern region serving a large area of an estimated catchment of 14 districts of Eastern Uganda composed of over 4 million people. The facility also offers many specialist services and it is a training ground for the Busitema University, Faculty of Health Sciences.

### Study population

The study targeted all adult diabetic patients. Known DM patients enrolled for routine care in the diabetic outpatient clinic of MRRH (a government-funded facility that offers free services), were recruited. Included adult diabetic patients (≥ 18 years), who consented to participate in this study and excluded those patients who were diagnosed with DM on the day of enrollment and those found to have diabetic emergencies (hypoglycemia or any hyperglycemic state).

### Sample size and Sampling procedure

A sample size of 374 was calculated using the Kish -Leslie formula [18], to determine the prevalence of biomarkers for CKD among DM patients within a confidence interval of 95%. Participants were conveniently recruited by the trained research assistants who informed them about the study and procedures involved, using both English and local languages. Those who consented and met the inclusion criteria participated in the study.

### Data collection methods

Each participant was assigned a specific identification code, and their records were extracted to help complete the questionnaire. Participants were reviewed by the research assistants and the principal investigator, who collected data using the World Health Organization (WHO) modified stepwise questionnaire [25], adopted and contextualized to the Ugandan context to collect data on demographics, the biophysical parameters, lifestyle, and social characteristics, disease-specific characteristics including; duration of DM, type of glycemic agent used, presence of any co-morbidity, and laboratory results from all study participants. 15 to 25 participants were interviewed on every data collection day, without interrupting routine patient care. Chart reviews were used to determine any prior diagnosis of hypertension or renal disease and those with documented diagnosis of hypertension and/or on treatment for hypertension were said to be hypertensive. A participant found with a systolic blood pressure (SBP) of ≥ 140mmHG and/or a diastolic blood pressure (DBP) of ≥ 90mmHG was diagnosed with co-morbid hypertension.

A midstream urine sample was collected in a sterile urine specimen container following instructions that were given to the participants by the research assistant. This sample was safely transferred to Pallisa General Hospital Laboratory, an accredited laboratory per the recognized international standards, ISO 15189 – 2012, where it was used for determining the Urine Albumin Creatinine Ratio (UACR). Patients found to have a UACR of ≥ 30mg/g and above were considered to have proteinuria (a biomarker for CKD) in line with the KDIGO guidelines. 30–299mg/g was categorized as microalbuminuria while those who had a UACR of 300mg/g and above were categorized as macroalbuminuria.

A venous blood sample was drawn from every participant by a qualified laboratory technician, from which several tests were done including; serum creatinine measured for all the study participants using a HumaStar 200 clinical chemistry machine, calibrated by the creatinine Jaffe 2 method. The results were used to calculate the eGFR using the CKD-EPI equation. Those with eGFR of ≤ 60mls/min/1.72m^2^ were considered to have a marker for CKD. The eGFR was also used for CKD staging as per KDIGO-CKD classification.

The other test conducted on the blood sample collected was a lipid panel and participants were said to have dyslipidemia if they had total cholesterol levels of ≥ 200mg/dl and/or High-Density Lipoprotein (HDL) of ≤ 40mg/dl. As is the routine practice in the DM clinic of MRRH, Fasting Blood Glucose (FBG) was measured for every participant using a Glucometer - SD CodeFree, Thailand. Those with an FBG of ≥ 7mmol/L were said to have poor glycemic control.

To determine the participants’ Basal Metabolic Index (BMI), their heights and weights were measured using a medical giraffe height measuring stadiometer (Model HMS PL) and an analog flat weighing scale respectively. Measurements were taken to the nearest 0.1 m and 0.1 kg for height and weight, respectively. BMI was computed and participants were categorized following the WHO BMI classification into underweight (< 18.5kg/m2), ideal weight (18.5– 24.9kg/m2), overweight (25.0–29.9 kg/m2), and obese (≥ 30 kg/m2).

### Data analysis

Our primary outcome was the prevalence of biomarkers for CKD (low eGFR ≤ 60mls/min/1.72m^2^ and/or proteinuria – UACR of ≥ 30mg/g) within the study population The predictor variables included; Lifestyle factors (smoking, inactivity, alcohol consumption), biophysical measurements (BMI), DM related clinical factors (duration of DM, hypoglycemic agents used, subjective adherence to the anti-diabetic drugs, glycemic control, dyslipidemia), non modifiable factors (age, sex, family history of renal disease) and co-morbidities (HIV status, hypertension among others). Raw data was entered into Microsoft Excel and analyzed using STATA version 17 (Stata Corp, College Station, TX). Categorical variables were described as proportions (%), and continuous variables were described as medians [interquartile range (IQR)]. In the univariate analysis, Fisher’s exact and chi-squared tests were used for categorical and binary variables respectively and the Wilcoxon rank-sum test was used for continuous variables. Predictors with a p-value < 0.05 were included in the multivariate model using logistic regression and the effect was measured using adjusted Odds Ratios (ORs).

## RESULTS

**Figure 1:**
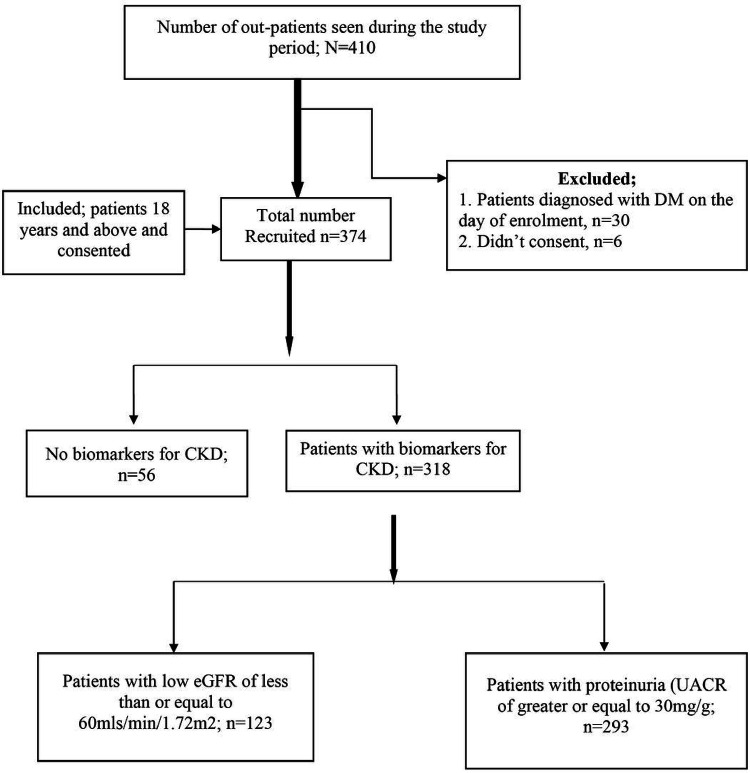
Study flow chart.

A total of 374 participants were recruited as shown in the study flow chart in Fig. 1 below;

### Socio-demographic characteristics of the patients:

The majority of the patients were females, 247 (66%), with a female-to-male ratio of 2:1. The median age of participants was 56, IQR of 13, the youngest participant was 22 years while the oldest participant was 90 years, see Table 1 below.

### Prevalence of biomarkers for CKD among diabetic patients attending the adult diabetic clinic of MRRH:

**Figure 2:**
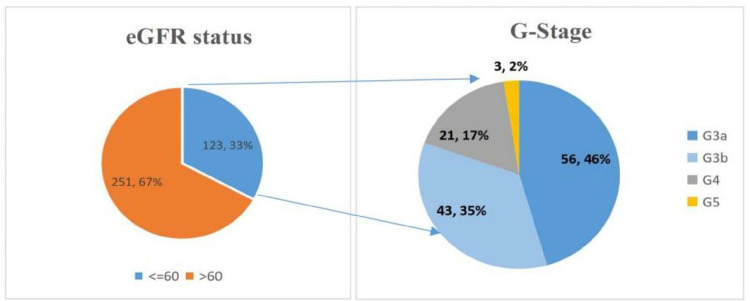
Prevalence of low eGFR among DM patients attending the adult diabetic clinic of MRRH.

The overall prevalence of biomarkers for CKD among diabetic patients attending the diabetic clinic was 85%. Of the 374 participants, 318 either had an estimated GFR of ≤ 60Ml/min/1.73m^2^, CKD defining proteinuria or both. There was a great discrepancy between the prevalence of low eGFR and that of proteinuria among the diabetic patients attending the adult diabetic clinic of MRRH. The prevalence of low eGFR among diabetic patients attending the diabetic clinic was 32.9% and among those with low eGFR, majority of them had their eGFR ≤ 44ml/min (54%), as shown in Fig. 2.

**Figure 3:**
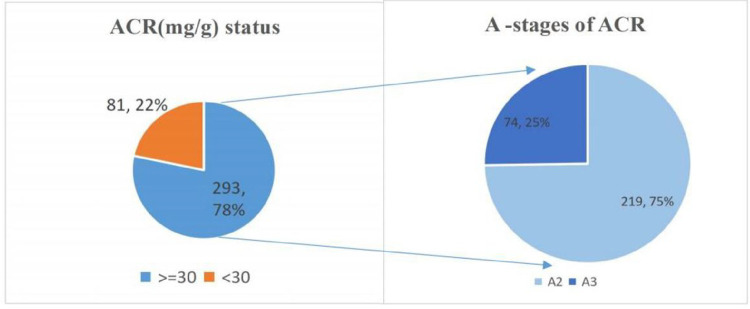
Prevalence of proteinuria among DM patients attending the adult diabetic clinic of MRRH.

The prevalence of proteinuria was 78.3%. Majority of those with proteinuria were among A2 (microalbuminuria) stage of the KDIGO proteinuria staging for CKD, as shown in Fig. 3.

### Factors associated with biomarkers for CKD among DM patients attending the adult diabetic out patients’ clinic of MRRH:

At univariate level of analysis, advanced age, duration of DM illness, subjective adherence to the antidiabetic drugs, presence of any co-morbidity, history of kidney disease, hypertension and dyslipidemia were found to be associated with the biomarkers for CKD in diabetic patients as shown in Table 2.

However, at multivariate analysis, only age, duration of DM, co-morbidity, hypertension and dyslipidemia were significantly associated with CKD biomarkers as shown in Table 3 below.

## DISCUSSION

Overall, in every 10 diabetic patients enrolled for care in the adult diabetic clinic of MRRH, nearly 8 had at least a biomarker for CKD. This outcome is consistent with the continental prevalence rate of CKD among diabetic patients on the African continent, estimated to vary from 11–83.7%, results from a systematic review of 32 similar studies [3]. And among those with low eGFR, a significant proportion, 54% fell under the category of advanced CKD, KDIGO G-stage ≥ G3b with 2% falling under stage 5 (End Stage Renal Disease, ESRD). Similarly, for those with proteinuria, a significant proportion, 25%, had macroalbuminuria (stage A-3) of CKD. Despite these alarming numbers of DM patients with biomarkers for CKD, a few (6.1%) were aware of their renal impairment. Thus, among diabetic patients, a significant number progress to advanced kidney disease without clinical or laboratory detection, which is a threat to the lives of such people, since the management of advanced kidney disease is still not readily affordable to a majority of Ugandans and is also associated with increased cardiovascular mortality and morbidity. This calls for the implementation of routine screening for CKD among diabetics in sub-Saharan Africa [26], a region where the screening for nephropathy among adult diabetics isn’t a routine practice due to limited resources [26,27,28].

Proteinuria prevalence is much higher than the other estimated prevalence within Uganda, almost doubling the 47.4% estimated prevalence from Muddu et al study (2014, 2015) [17], and almost tripling the 17.1% estimated prevalence among diabetic patients from Nambuya et al study (1996) [16]. This could be accounted for by the rising prevalence of NCDs over time, which has been projected to be more in developing countries like Uganda. The two studies quoted above were conducted close to a decade ago, hence the change in the prevalence estimates. The other explanation for the huge difference in the proteinuria prevalence estimate could be because of regional variations in the prevalence of CKD, the above-quoted studies having been conducted in central Uganda, plus a few more studies conducted in western Uganda, which could yield different prevalence and this may be attributed to the geographical, cultural, dietary as well as lifestyle differences.

Advanced age, duration of DM illness, co-morbid hypertension, and dyslipidemia were significantly associated with biomarkers for CKD in our study population. This has been a consistent finding in other studies [1,3,4,8,9,10,11,20]. Age is an independent risk factor for most cardiovascular diseases and with advancing age, there is an associated loss of nephron mass [29]. The longer the time of exposure to hyperglycemia by the glomerular structures, the greater the risk of structural damage of the glomerulus as a result of the formation of advanced glycated end products that result from non-enzymatic glycation of the endothelial structures [22]. Hypertension independently contributes to hypertensive nephropathy via glomerulosclerosis due to persistent high blood pressure [23]. However, hypertension could also result from chronic kidney disease following the activation of the RAAS, among other mechanisms [24]. Dyslipidemia is one of the known risk factors for cardiovascular disease, but similarly, CKD in advanced stages can be complicated by dyslipidemia, a mechanism attributed to the persistent and excessive proteinuria that occurs in Diabetic kidney disease leading to a compensatory effect by the liver resulting into excessive synthesis of lipoproproteins which are involved in the synthesis of lipids thus causing hyperlipidemias [30].

Unlike other studies, our study didn’t find a significant association between obesity and overweight to biomarkers for CKD. Most studies established that DM patients with obesity had higher prevalence rates of CKD as determined by the low eGFR and proteinuria; CREDO study [1], and many other similar studies; Ekoru et al, Low & Chi Lim et al, Middleton et al, Sabanayagam et al [8,9,10,11]. Among our study participants, the majority had normal weight (50.8%), the overweight category being 33.4%, only 12.6% were obese and another 3.2% were underweight. These findings are consistent with the findings of Kibirige’s study [21] which identified that, compared to the Western world; the majority of patients with DM in Africa are younger and relatively lean in body size.

### Study strengths;

This study looked at low eGFR, proteinuria, or both biomarkers to arrive at the overall prevalence, compared to several similar studies that used single entities to estimate CKD prevalence.

The research provides potential information in the development of a clinical protocol for routine care for DM patients, to cater for routine screening for CKD biomarkers as well as timely assessment and treatment of renal impairment in patients with DM.

### Limitations of the study;

Being a cross-sectional study design, we did not determine what came first, whether it was DM or CKD biomarkers, and for the participants who had both DM and hypertension, we could not determine which disease condition was responsible for the biomarkers for CKD.

HBA1C could have been the preferred predictor for glycemic control, but our study was limited by finances thus we relied on fasting blood sugar, a weaker predictor for longer-term glycemic control.

During enrollment of study participants, those on ACEIs were not excluded, which could underscore the outcome of proteinuria.

## CONCLUSION

The prevalence of biomarkers for CKD among adult diabetic patients is quite high compared to the global and national prevalence of CKD in the general population. This remains a threat to the life span of the diabetic population as well as the quality of life of these patients, yet only a handful of these patients are aware of their impaired kidney functioning, implying that the majority of patients with biomarkers for CKD among diabetic patients are not aware of their condition and thus not on any measures to help slow down the progression of kidney disease. The other key observation drawn from our study is that a significant proportion of diabetic patients had markers of severe kidney disease, with 2% falling within ESRD, eGFR ≤ 15mls/min/1.72m^2^. We therefore recommend that; periodic screening for CKD, among diabetic patients should be readily incorporated into the routine care package, within periods of 3, 6 months, or annually as guided by the individual patient characteristics. Routine care for diabetic patients should emphasize screening and managing co-morbidities including hypertension, and dyslipidemia.

## Figures and Tables

**Table 1:** Socio-demographic characteristics of the patients.

Variable	Frequency n = 374	Percentage (%)
**Age (years)**		
22–39	27	7.2
40–59	203	54.3
60+	144	38.5
**Sex**		
Female	247	66.0
Male	127	34.0
**Religion**		
Moslem	133	35.6
Christian	241	64.4
**Residence**		
Urban	169	45.2
Rural	205	54.8
**Marital status**		
Single	17	4.5
Married/cohabiting	254	67.9
Divorced/separated	35	9.4
Widowed	68	18.2
**Level of education**		
None	68	18.2
Primary	183	48.9
Secondary	90	24.1
Post-secondary	33	8.8
**Occupation**		
Formal employment	61	16.4
Informal employment	311	83.6
**Duration of DM illness(years)**		
< 1	47	12.6
1–5	174	46.5
6–10	84	22.5
> 10	69	18.5

**Table 2:** Factors associated with CKD biomarkers among diabetic patients attending the adult diabetic clinic inMRRH.

Variable	Total n = 374	Biomarkers for CKD		P-value
		Absent n = 56(15)	Present n = 318(85)	
**Age (years)**				0.019
22–39	27(7.2)	20(74.1)	7(25.9)	
40–59	203(54.3)	30(14.8)	173(85.2)	
60+	144(38.5)	6(4.2)	138(95.8)	
**Sex**				0.756
Female	247(66.0)	38(15.4)	209(84.6)	
Male	127(34.0)	18(14.2)	109(85.8)	
**Residence**				0.929
Urban	169(45.2)	25(14.8)	144(85.2)	
Rural	205(54.8)	31(15.1)	174(84.9)	
**Level of education**				0.290
No formal education	68(18.2)	15(22.1)	53(77.9)	
Primary	183(48.9)	23(12.6)	160(87.4)	
Secondary	90(24.1)	14(15.6)	76(84.4)	
Post-secondary	33(8.8)	4(12.1)	29(87.9)	
**Occupation**				0.434
Formal employment	61(16.4)	11(18)	50(82)	
Informal employment	311(83.6)	44(14.1)	267(85.9)	
**BMI**				0.841
Underweight	12(3.2)	2(16.7)	10(83.3)	
Normal	190(50.8)	28(14.7)	162(85.3)	
Overweight	125(33.4)	19(15.2)	106(84.8)	
Obese	47(12.6)	7(14.9)	40(85.1)	
**Duration of DM illness (years)**				0.018
< 1	47(12.6)	17(36.2)	30(63.8)	
1–5	174(46.5)	29(16.7)	145(83.3)	
6–10	84(22.5)	6(7.1)	78(92.9)	
> 10	69(18.5)	4(5.2)	65(94.2)	
**Current DM treatment**				0.528
Non-pharmacological	17(4.5)	1(5.9)	16(94.1)	
Oral hypoglycemic drugs	250(66.8)	41(16.4)	209(83.6)	
Insulin	54(14.4)	9(16.7)	45(83.3)	
Herbal remedies	6(1.6)	1(16.7)	5(83.3)	
Both insulin and oral hypoglycemic drugs	47(12.6)	4(8.5)	43(91.5)	
**Adherence to the anti-diabetic drugs**				0.013
Takes the right treatment and on time as advised by the health worker	296(79.1)	45(15.2)	251(84.8)	
Doesn’t take treatment as advised by the health worker	68(18.2)	1(1.5)	67(98.5)	
Missing	10(2.7)			
**Medical history of kidney disease**				0.032
Yes	23(6.1)	7(30.4)	16(69.6)	
No	351(93.9)	49(14)	302(86)	
**Any comorbidity**				0.009
Yes	350(93.6)	48(13.7))	302(86.3)	
None	24(6.4)	8(33.3)	16(66.7)	
**Hypertension**				0.004
Known hypertensive	209((55.9)	2(1)	207(99)	
Not known hypertensive	165(44.1)	54(32.7)	111(67.2))	
**Blood pressure at enrolment**				0.305
High BP	176(47.1)	6(3.4)	170(96.6)	
Normal	198(52.9)	50(25.3)	148(74.7)	
**Family history**				
DM	143(38.2)	16(11.2)	127(88.8)	0.107
Kidney disease	28(7.5)	4(14.3)	24(85.7)	0.916
Obesity	70(18.7)	7(10)	63(90)	0.196
**Smoking**				0.323
Yes	8(2.1)	0(0.0)	8(100)	
Stopped	34(9.1)	22(64.7)	12(35.3)	
Never smoked	332(88.8)	34(10.2)	298(89.8)	
**Alcohol**				0.691
Yes	39(10.4)	5(12.8))	34(87.2)	
Never taken	321(85.8)	37(11.5)	284(88.5)	
Missing	14(3.7)			
**Physical activity**				0.309
Regular exercise	231(61.8)	38(16.5)	193(83.5)	
Doesn’t exercise regularly	143(38.2)	18(12.6)	125(87.4)	
**FBG**				0.867
< 7mmol	144(38.5)	4(2.8)	140(97.2)	
≥ 7mmol	230(61.5)	52(22.6)	178(77.4)	
**Lipid profile**				0.006
Dyslipidemia	197(52.7)	2(1)	195(99)	
Normal lipid panel	177(47.3)	52(29.4)	125(70.6)	

**Table 3:** Odds ratios for the factors associated with CKD biomarkers among diabetic patients of MRRH.

Variable	COR (95% CI)	P-value	AOR (95% CI)	P-value
**Age (years)**				
22–39	1		1	
40–59	1.5(0.6, 3.9)	0.405	1.7(0.6, 4.5)	0.286
60+	2.8(1.1, 7.3)	0.035	3.1(1.2, 8.4)	0.023
**Duration of DM illness**				
Less than 1 year	1		1	
1–5 years	1.1(0.8,1.8)	0.092	1(0.9,1.5)	0.208
6–10 years	1.4(1.1,1.9)	0.034	1.2(0.9,1.7)	0.094
More than 10 years	1.8(1.5,2.2)	0.006	1.5(1.2,1.8)	0.032
**Comorbidity**				
DM alone	1		1	
Any comorbidity	1.8(1.4,2.4)	0.003	1.6(1.2,2.0)	0.042
**Adherence**				
Follows given instructions	1		1	
Doesn’t follow given instructions	1.8(1.6,2.4)	0.006	1.6(1.2,2.6)	0.054
**Hypertension**				
Not known hypertensive	1		1	
Known	2.2(1.8,2.8)	0.001	1.8(1.6,2.0)	0.031
**Lipid panel**				
Normal lipid panel	1		1	
Dyslipidemia	2.6(1.3, 4.8)	0.001	2.4(1.4, 4.2)	0.001

## Data Availability

The datasets used during our study are available from the corresponding author on reasonable request.
